# Effects of Microtubule Stabilization by Epothilone B Depend on the Type and Age of Neurons

**DOI:** 10.1155/2016/5056418

**Published:** 2016-10-31

**Authors:** Eun-Hae Jang, Aeri Sim, Sun-Kyoung Im, Eun-Mi Hur

**Affiliations:** ^1^Center for Neuroscience, Brain Science Institute, Korea Institute of Science and Technology (KIST), Seoul, Republic of Korea; ^2^Department of Neuroscience, Korea University of Science and Technology, Daejeon, Republic of Korea; ^3^Convergence Research Center for Diagnosis, Treatment and Care System of Dementia, Korea Institute of Science and Technology (KIST), Seoul, Republic of Korea

## Abstract

Several studies have demonstrated the therapeutic potential of applying microtubule- (MT-) stabilizing agents (MSAs) that cross the blood-brain barrier to promote axon regeneration and prevent axonal dystrophy in rodent models of spinal cord injury and neurodegenerative diseases. Paradoxically, administration of MSAs, which have been widely prescribed to treat malignancies, is well known to cause debilitating peripheral neuropathy and axon degeneration. Despite the growing interest of applying MSAs to treat the injured or degenerating central nervous system (CNS), consequences of MSA exposure to neurons in the central and peripheral nervous system (PNS) have not been thoroughly investigated. Here, we have examined and compared the effects of a brain-penetrant MSA, epothilone B, on cortical and sensory neurons in culture and show that epothilone B exhibits both beneficial and detrimental effects, depending on not only the concentration of drug but also the type and age of a neuron, as seen in clinical settings. Therefore, to exploit MSAs to their full benefit and minimize unwanted side effects, it is important to understand the properties of neuronal MTs and strategies should be devised to deliver minimal effective concentration directly to the site where needed.

## 1. Introduction

Microtubules (MTs) are essential for a wide range of dynamic cellular processes in the nervous system. MTs control neuronal migration, differentiation, and formation of synaptic connections during development and continue to play essential roles in the mature nervous system by providing architectural support and serving as major tracks for anterograde and retrograde transport of organelles and other cargos. Indeed, nerve injury causes disorganization of MTs [1] and perturbation of MT dynamics is a common feature in a number of neurodevelopmental [2] and neurodegenerative diseases [3].

In recent years, MTs have emerged as a promising target for treating injury and disease of the central nervous system (CNS) [4, 5]. In particular, it has been suggested that strategies that stabilize MTs would prevent the breakdown or degeneration of MTs after injury or in neurodegenerative diseases and might even strengthen MT arrays in a manner to induce repair and regeneration of the damaged connections [6, 7]. Applying MT-stabilizing agents (MSAs) to treat injury and diseases in the CNS has become more feasible by the development of MSAs that cross the blood-brain barrier (BBB), such as epothilones. In a rodent model of spinal cord injury, systemic administration of epothilone reduced scar formation and promoted axon regeneration [8]. Moreover, intraperitoneal administration of epothilone suppressed axonal MT loss and alleviated cognitive defects in a mouse model of tauopathy [9, 10] and systemic injection of epothilone was neuroprotective in a rodent model of Parkinsonism [11]. These experiments suggest a strong potential for applying brain-penetrant MSAs to promoting axon regeneration in the CNS and improving pathological and functional outcomes in neurodegenerative diseases.

Various derivatives of MT-stabilizing agents (MSAs) comprise chemotherapeutic drugs that are routinely prescribed for the treatment of several types of malignancies [12]. By stabilizing MTs, MSAs prevent the requisite dynamics of MTs during cell cycle progression, leading to mitotic arrest, and ultimately apoptosis. Thus, MSAs are highly effective against proliferating cancer cells. Paradoxically, neurons, which are postmitotic and have long been thought to have stable MTs [13–15], are also susceptible to MSA-based chemotherapy regimens. Indeed, a major adverse effect associated with MSA-based chemotherapy is peripheral neuropathy, which can lead to cessation of treatment and sometimes cause lasting effects, deteriorating the quality of life of patients with cancer [16]. Peripheral sensory neurons seem to be especially sensitive to MSA exposure [17]. It has been speculated that the hypersensitivity in patients might be related with the amount of exposure, that is, sensory DRG neurons lie outside of the BBB and are supplied with fenestrated capillaries that allow free passage of circulating drugs [18, 19], whereas neurons in the CNS are preserved, presumably because most drugs have poor BBB permeability [17]. According to this conjecture, it is possible that MSAs that penetrate BBB cause CNS toxicity. Alternatively, peripheral neuropathy might be due to the intrinsic vulnerability of MTs assembled in sensory neurons. Despite the increasing interest of applying MSAs to treat the injured or degenerating nervous system, consequences of MSA exposure to CNS and PNS neurons have not been thoroughly investigated. Here we have aimed at investigating and comparing the effects of epothilone B, which is a brain-penetrant MSA, in various types of neurons in culture.

## 2. Materials and Methods

### 2.1. Animals

All experiments involving animals were performed in accordance with the guidance of the Institutional Animal Care and Use Committee of the Korea Institute of Science and Technology. Ten- to twelve-week-old female ICR mice (weighing from 30 to 35 g) were purchased from DBL and housed in the Korea Institute of Science and Technology Animal Facility.

### 2.2. Primary Cell Culture and Drug Treatment

#### 2.2.1. Embryonic Cortical Neurons

**Figure 1:**
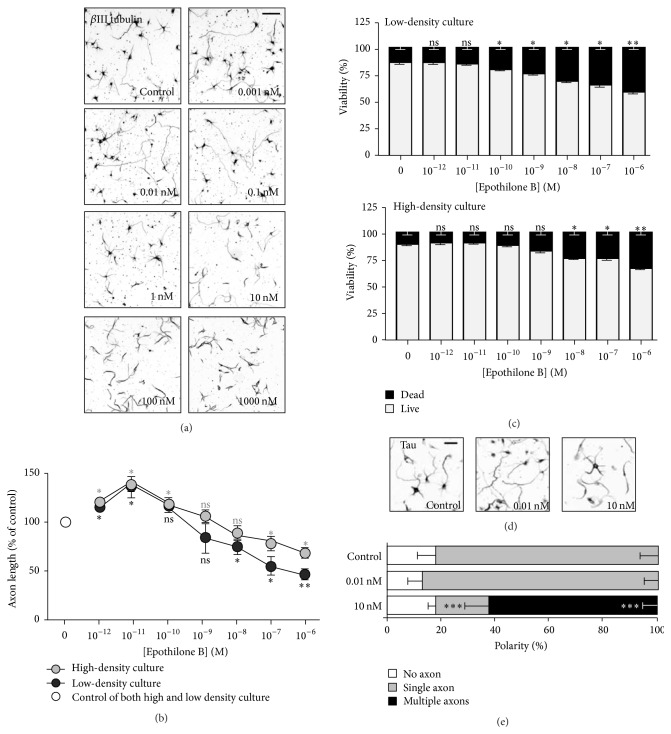
Effects of epothilone B on axon growth and cell viability in embryonic cortical neurons. (a–e) Embryonic day 15.5 (E15.5) cortical neurons were plated at a low density (5 × 10^4^ cells/1.9 cm^2^) or at high density (2 × 10^5^ cells in 1.9 cm^2^) and cultured in the absence or presence of various concentrations of epothilone B, from 1 pM to 1 *μ*M, as indicated. Low- and high-density cultures were fixed at 72 hr and 24 hr after plating, respectively, and subjected to immunostaining with anti-Tuj1 antibodies (a, b). Representative images ((a), inverted images from Tuj1 immunostaining; low-density culture) and quantification of axon length (b) are shown (gray, high-density culture and black, low-density culture). Cell viability was assayed by propidium iodide (PI) staining (c). To assess polarization, neurons treated with vehicle (control) or the indicated concentration (0.01 nM or 10 nM) of epothilone B were immunostained with anti-Tau antibodies (d, e). Representative images (d) and quantification of neuronal polarity (e) are shown. Scale bar, 50 *μ*m. ^*∗*^ *p* < 0.05, ^*∗∗*^ *p* < 0.01, and ^*∗∗∗*^ *p* < 0.001; ns, statistically not significant.

Cortices were dissected from embryonic 15.5 mice and incubated in papain (20 U/mL, Worthington) containing DNase (10 U/*μ*L, Sigma) for 40 min at 37°C. Enzyme-digested cortices were washed three times with MEM (Cellgro) containing 10% heat inactivated fetal bovine serum (Hyclone) and dissociated in culture medium. Dissociated neurons were then centrifuged to remove the supernatant and counted cells were plated on glass coverslips coated with 100 *μ*g/mL poly-D-lysine (Sigma) and grown in Neurobasal A (Gibco) medium containing Glutamax (1%, Thermo Fisher Scientific) and B27 (2%, Thermo Fisher Scientific) supplements. 5 hr after plating, neurons were treated with epothilone B (from 1 pM to 1 *μ*M in Figure 1 and 10 pM or 1 nM in Figure 4) for 3 days in low-density cultures (5 × 10^4^ cells in 1.9 cm^2^) or overnight (17–19 hr) in high-density cultures (2 × 10^5^ cells in 1.9 cm^2^). Neurons were then fixed and analyzed for viability, axon growth, and microtubule structure. For nocodazole treatment, neurons were cultured for 3 days to allow differentiation and then exposed to either 50 nM for 1.5 hr (Figure 5) or 1 *μ*M for 30 min (Figure 6), right before fixation.

#### 2.2.2. Embryonic and Postnatal Dorsal Root Ganglion (DRG) Neurons

**Figure 2:**
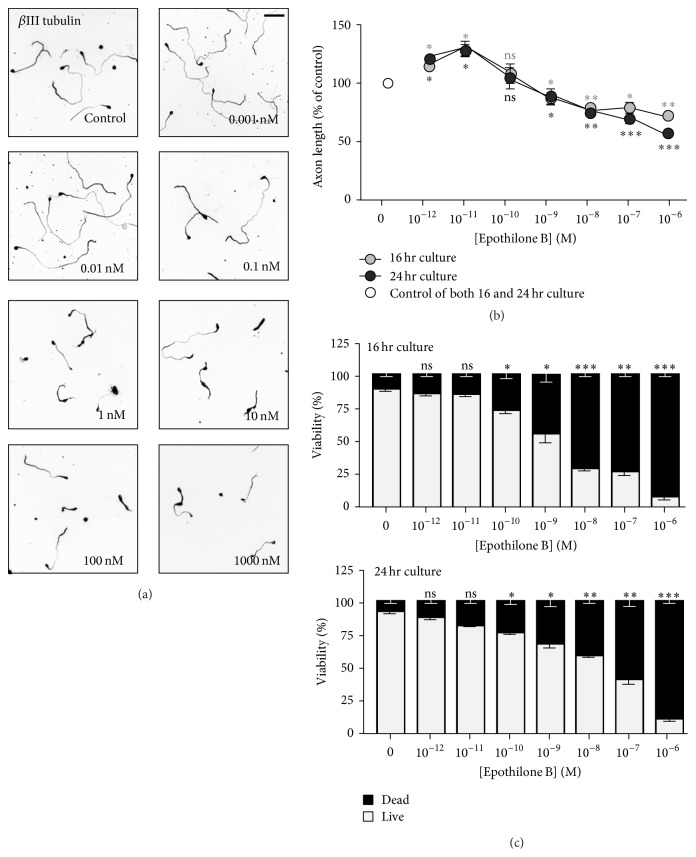
Effects of epothilone B on axon growth and cell viability in embryonic dorsal root ganglion (DRG) neurons. (a–c) Embryonic day 13.5 (E13.5) DRG neurons were plated at a low density and cultured in the absence or presence of various concentrations of epothilone B, from 1 pM to 1 *μ*M, as indicated. Neurons were fixed after overnight culture (fixed at 16 hr or 24 hr after plating) and immunostained with anti-Tuj1 antibodies. Representative images ((a), inverted images from Tuj1 immunostaining (16 hr culture) and quantification of axon length (b) are shown (gray, 16 hr culture and black, 24 hr culture). Cell viability was assayed by PI staining (c). Scale bar, 100 *μ*m. ^*∗*^ *p* < 0.05, ^*∗∗*^ *p* < 0.01, and ^*∗∗∗*^ *p* < 0.001; ns, statistically not significant.

**Figure 3 fig3:**
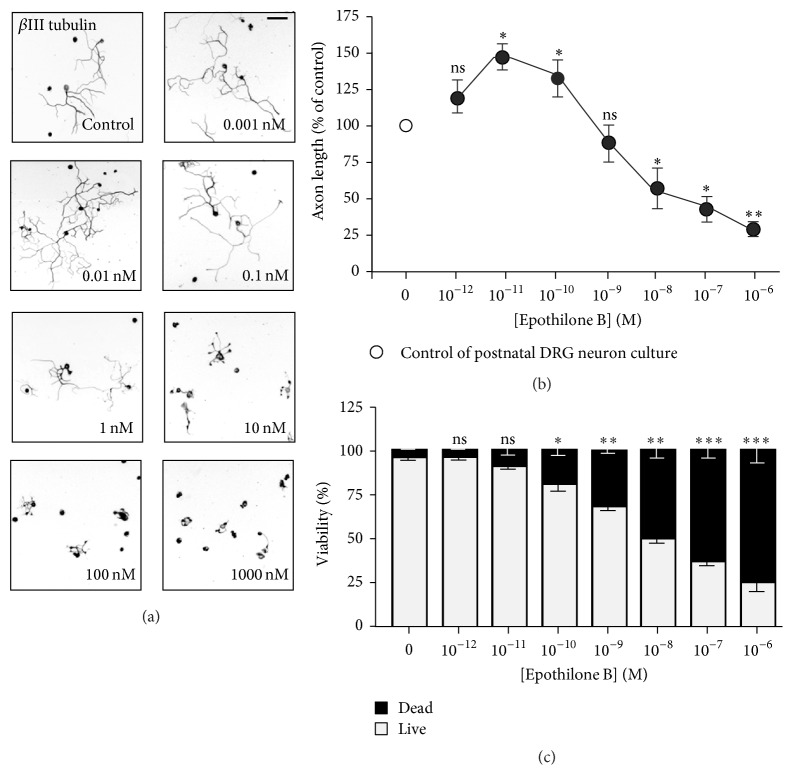
Effects of epothilone B on axon growth and cell viability in postnatal DRG neurons. (a–c) Postnatal day 4 (P4) DRG neurons were cultured in the absence or presence of various concentrations of epothilone B, from 1 pM to 1 *μ*M, as indicated. Neurons were fixed at 24 hr after plating and immunostained with anti-Tuj1 antibodies. Representative images ((a), inverted images from Tuj1 immunostaining) and quantification of axon length (b) are shown. Cell viability was assayed by PI staining (c). Scale bar, 100 *μ*m. ^*∗*^ *p* < 0.05, ^*∗∗*^ *p* < 0.01, and ^*∗∗∗*^ *p* < 0.001; ns, statistically not significant.

**Figure 4:**
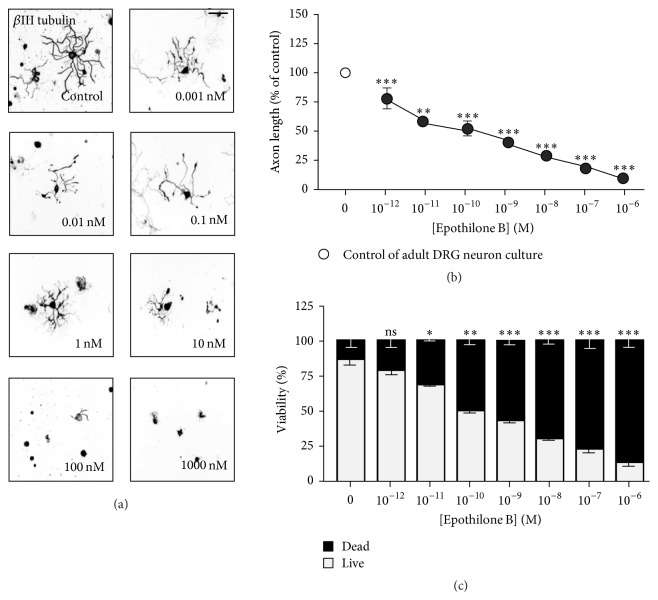
Effect of epothilone B on axon growth and cell viability in adult DRG neurons. (a–c) Adult DRG neurons were plated at a low density and cultured in the absence or presence of various concentrations of epothilone B, from 1 pM to 1 *μ*M, as indicated. Neurons were fixed at 24 hr after plating and immunostained with anti-Tuj1 antibodies. Representative images ((a), inverted images from Tuj1 immunostaining) and quantification of axon length (b) are shown. Cell viability was assayed by PI staining (c). Scale bar, 100 *μ*m. ^*∗*^ *p* < 0.05, ^*∗∗*^ *p* < 0.01, and ^*∗∗∗*^ *p* < 0.001; ns, statistically not significant.

**Figure 5:**
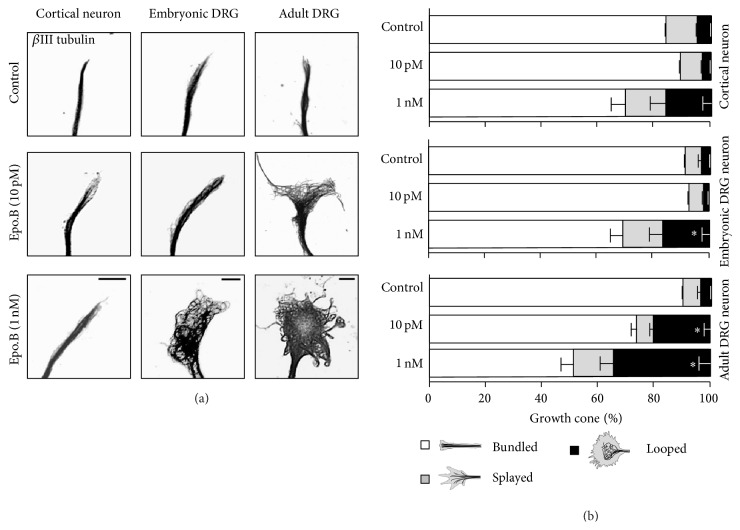
Epothilone B induces changes in growth cone MT structure. (a-b) Embryonic (E15.5) cortical neurons, embryonic (E13.5) DRG neurons, or adult DRG neurons were cultured in the absence or presence of epothilone B (either 10 pM or 1 nM) and were fixed, followed by immunostaining with anti-Tuj1 antibodies. Exposure time has been adjusted for each image to optimize the visibility of the MT network. Representative images (a) and quantifications of growth cone MT network (b) are shown. Scale bar, 10 *μ*m. ^*∗*^ *p* < 0.05, statistically not significant.

**Figure 6:**
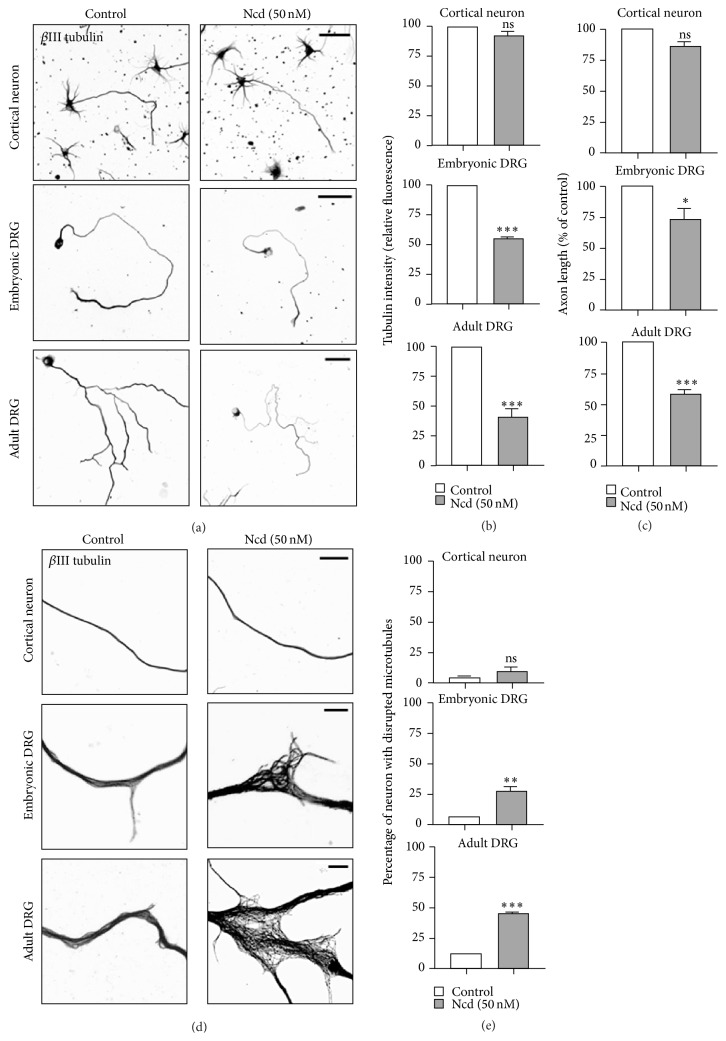
Neurons of different type and age contain MTs that differ in stability. (a–d) Embryonic (E15.5) cortical neurons, embryonic (13.5) DRG neurons, or adult DRG neurons were cultured in the absence or presence of nocodazole (Ncd) (50 nM, 1.5 hr) and were immunostained with anti-Tuj1 antibodies. Representative images (a, d) and quantification of fluorescence intensity of *β*III-tubulin immunostaining (b), axon length (c), and percentage of neurons with disrupted MTs (e) are shown. Scale bar, 50 *μ*m. ^*∗*^ *p* < 0.05, ^*∗∗*^ *p* < 0.01, and ^*∗∗∗*^ *p* < 0.001; ns, statistically not significant.

DRGs were dissected from either embryonic day 13.5 (E13.5) or postnatal day 4 (P4) mice. Embryonic DRG neurons were digested in collagenase A (1 mg/mL, Roche) for 15 min, followed by trypLE (0.05%, Life Technologies) for 5 min at 37°C and postnatal DRG neurons were digested in collagenase A (1 mg/mL) for 25 min, followed by trypLE (0.05%) for 7 min at 37°C. Enzyme-digested embryonic and postnatal DRGs were washed three times with MEM containing 10% fetal bovine serum and dissociated in cultured medium containing NGF (25 ng/mL, Alomone labs). Dissociated neurons were then plated on glass coverslips coated with a mixture of 100 *μ*g/mL poly-D-lysine and 10 *μ*g/mL laminin (Gibco) and grown in MEM containing NGF (25 ng/mL, Alomone labs) and antibiotics (20 *μ*M 5-fluoro-2-deoxyuridine and 20 *μ*M uridine). 5 hr after plating, neurons were treated with epothilone B (from 1 pM to 1 *μ*M in Figures 2 and 3 and 10 pM or 1 nM in Figure 4) overnight (11 hr treatment for Figure 2(a); either 11 hr or 17–19 hr treatment for Figures 2(b) and 2(c); and 19 hr treatment for Figure 3). Neurons were then fixed and analyzed for viability, axon growth, and microtubule structure. For nocodazole treatment, neurons were cultured overnight to allow axon growth and then exposed to either 50 nM for 1.5 hr (Figure 5) or 1 *μ*M for 30 min (Figure 6), right before fixation.

#### 2.2.3. Adult Dorsal Root Ganglion (DRG) Neurons

DRGs were dissected from ten- to twelve-week-old adult mice and digested in collagenase A (1 mg/mL) for 90 min, followed by trypLE (0.05%) for 25 min at 37°C. Enzyme-digested DRGs were washed three times with MEM containing 10% fetal bovine serum and dissociated in cultured medium. Dissociated neurons were then centrifuged to remove the supernatant and plated on glass coverslips coated with a mixture of 100 *µ*g/mL poly-D-lysine and 10 *μ*g/mL laminin and grown in MEM containing antibiotics (20 *μ*M 5-fluoro-2-deoxyuridine and 20 *μ*M uridine) without the addition of any growth factors. 5 hr after plating, neurons were treated with epothilone B (from 1 pM to 1 *μ*M in Figure 3 and 10 pM or 1 nM in Figure 4) overnight (17–19 hr) and then fixed and analyzed for viability, axon growth, and microtubule structure. For nocodazole treatment, neurons were cultured overnight to allow axon growth and then exposed to either 50 nM for 1.5 hr (Figure 5) or 1 *μ*M for 30 min (Figure 6), right before fixation.

### 2.3. Immunostaining and Fluorescence Microscopy

Neuronal microtubules were stained as described elsewhere [20, 21]. Briefly, neurons were simultaneously fixed and detergent extracted in a solution containing prewarmed 4% paraformaldehyde (PFA), 0.15% glutaraldehyde, and 0.2% Triton X-100 dissolved in PBS (37°C, 20 min). Fixed neurons were blocked in a blocking solution (2% BSA, 0.2% Triton X-100 in PBS). Primary (beta III tubulin, T8578, Sigma Aldrich, 1 : 2000; Tau, number 9632, Cell Signaling, 1 : 1000) and secondary antibodies (Alexa fluor 488, A11029, Invitrogen) were diluted in the blocking buffer. All secondary antibodies (1 : 400–500) were incubated for 1 hr at room temperature. After extensive rinsing with PBS, coverslips were mounted onto glass slides for observation. All coverslips in any one experiment were fixed and processed together. Neurons were viewed under an inverted light microscope (Axio Observer Z1, Carl Zeiss MicroImaging, Inc.) equipped with epifluorescence optics. Images were captured with a CCD camera controlled by the ZEN software (Carl Zeiss MicroImaging, Inc. A 5x, a 10x, or a 20x objective (0.45 NA)) was used to record whole neurons. A 100x (Confocal, 1.3 NA) oil objective was used for high-resolution imaging of growth cone microtubule structures.

### 2.4. Measurement of Axon Growth

All images analyses were performed using the “measure/curve” application of ZEN (Carl Zeiss MicroImaging, Inc.) and Image J (win 32). For quantification of axon length, neurons processing neurites longer than one-cell diameter were photographed and the longest axon of each neuron was traced manually. In each experiment, at least 60 neurons per condition were measured unless stated otherwise to calculate the mean value. At least three independent experiments were performed to compute the mean axon length and s.e.m values, which were used to plot the bar graphs.

### 2.5. Neuron Viability Assay

Neuronal viability was assessed by staining the cells with 10 *µ*g/mL propidium iodide (PI; Sigma) for 20 min at 37°C. PI-labeled cells were fixed and immunostained for neuronal tubulin to identify both healthy and dead cells. Five microscope fields (5x) were randomly chosen from each culture well and photographed. Live/dead cells were presented as percentage of total cells.

### 2.6. Statistics

Presented images are representative of at least three independent experiments and all data were calculated from at least three independent experiments. Data are presented as mean ± s.e.m. Two-tailed Student's* t*-test was used to determine the statistical significance between different experimental groups, which was set at a value of *p* < 0.05.

## 3. Results

### 3.1. Effects of MT Stabilization by Epothilone B on Embryonic Cortical Neurons

To investigate and compare the effects of epothilone B in various types of neurons, we first examined how neurite outgrowth and cell viability were affected in embryonic cortical neurons. Embryonic cortical neurons were plated at low density (5 × 10^4^ cells in 1.9 cm^2^) in order to obtain singly isolated neurons with clearly identifiable and measurable axons. In low-density cultures, neurons underwent polarization and developed into stage 3 neurons in three days. When observed at DIV3, we found that epothilone B exhibited both axon growth-promoting and growth-inhibiting effects, depending on the concentration (Figures 1(a) and 1(b)). Epothilone markedly promoted axon growth at picomolar concentrations, whereas it prevented axon growth at nanosubmicromolar concentrations (10–1000 nM). Propidium iodide (PI) staining revealed that epothilone B reduced cell viability in a dose-dependent fashion and cell death became evident at 100 pM and higher (Figure 1(c)). We also observed that cortical neurons treated with 10 nM or higher concentrations of epothilone developed multiple axons, whereas 10 pM had no effect on neuronal polarity (Figures 1(d) and 1(e)).

When neurons were plated at higher density (2 × 10^5^ cells in 1.9 cm^2^), cortical neurons underwent polarization and developed into stage 3 neurons within a day. Treatment with various concentrations of epothilone B in higher density cultures for one day produced similar results (Figures 1(b) and 1(c)) as in the low-density 3-day culture, inducing both axon growth-promoting and growth-inhibiting effects depending on the concentration. However, the inhibitory effects on axon growth and cell viability were reduced. In high-density cultures, cell death became statistically significant at 10 nM or higher (Figure 1(c)).

### 3.2. Cytotoxic Effect of Epothilone B on DRG Neurons

To investigate the effects of epothilone B in neurons in the PNS, we isolated and cultured dorsal root ganglion (DRG) neurons from embryos, neonates, and adult mice and treated the neurons with various concentrations of epothilone B. Similar to cortical neurons, in embryonic and postnatal DRG neurons, epothilone B exhibited both axon growth-promoting and growth-inhibiting effects, depending on the concentration (Figures 2(a), 2(b), 3(a), and 3(b)), and the dual effect was more prominent in postnatal neurons. At 10 pM, axon growth was promoted to 29.5 ± 7.2% and 46.5 ± 6.7% in embryonic and postnatal neurons, respectively, whereas, at 1 *μ*M, growth was inhibited to 27.4 ± 2.6% and 70.5 ± 6.3% in embryonic and postnatal neurons, respectively. When cell viability was assessed by PI staining, we found that embryonic and postnatal DRG neurons were much more sensitive to epothilone treatment (Figures 2(c) and 3(c)) as compared to embryonic cortical neurons (see Figure 1(c)). In embryonic and postnatal DRG neurons, cell death was evident from 100 pM, and, at 1, the vast majority of neurons failed to survive (89.1 ± 2.5% and 75.5 ± 6.9% death in embryonic and postnatal DRG neurons, resp., versus 31.1 ± 1.6% death in cortical neurons).

In striking contrast, we found that in adult DRG neurons, epothilone B markedly prevented axon growth at all concentrations tested (Figures 4(a) and 4(b)). Even at 10 pM, the concentration which induced a maximum axon growth-promoting effect in embryonic and postnatal DRG neurons (see above), axon growth was drastically inhibited in adult sensory neurons (41.8 ± 2.0% inhibition). PI staining also revealed severe toxicity of epothilone B in adult DRG neurons (Figure 4(c)). Together, these results clearly show that (i) epothilone can induce a dual effect on axon growth, (ii) whether epothilone produces a growth-promoting or a growth-inhibiting effect depends on not only its concentration but also the type and age of a neuron being exposed to the drug, and (iii) peripheral sensory neurons exhibit higher susceptibility to epothilone exposure compared to cortical neurons.

### 3.3. Disruption of MT Configuration in the Growth Cone

As the tightly bundled axonal MTs enter the growth cone, they display distinct structures, such as bundled, splayed, or looped configurations. Bundled MTs are found in rapidly advancing growth cones, whereas splayed or looped configurations are observed when growth cones are in a paused state [22]. To further compare the changes induced by epothilone B in different types of neurons, we cultured cortical and sensory neurons on polylysine laminin and examined how epothilone affected the configuration of growth cone MTs. It has been reported that when cultured on low concentration of laminin (5 *μ*g/mL laminin), growth cones from embryonic and adult DRG neurons display different morphologies [21]. Under such condition, growth cones of embryonic DRG neurons are larger and about 50% of growth cones are splayed, whereas the majority of adult neurons are tipped with small growth cones [21]. However, in our cultures in which we applied high concentration of laminin (10 *μ*g/mL laminin), growth cone morphologies of embryonic and adult DRG neurons were similar in the control condition. Most growth cones contained straight and tightly bundled MTs with their tips pointing toward the leading edge (91.2 ± 0.7% and 90.4 ± 1.2% of embryonic and adult growth cones, resp.).

Based on the axon growth profiles in response to various concentrations of epothilone B (see Figures 1(b), 2(b), and 4(b)), we tested the effects of 10 pM and 1 nM of epothilone B. In embryonic cortical and DRG neurons, growth cone MTs remained tightly bundled in response to 10 pM of epothilone (Figures 5(a) and 5(b)), consistent with the axon growth-promoting effect of 10 pM in those neurons (see Figures 1(b) and 2(b)). By contrast, when adult DRG neurons were exposed to the same concentration of epothilone, growth cone MTs became disorganized, often forming loops (Figures 5(a) and 5(b)), which was compatible with the growth-inhibiting effect of 10 pM in adult sensory neurons (Figure 4(b)). At 1 nM, we observed a tendency for growth cone MTs to splay or form loops, although the effect was not statistically significant, in embryonic cortical neurons, where the same concentration did not cause any noticeable effect on axon growth (Figure 1(b)). However, 1 nM changed MT configuration in embryonic and adult DRG neurons (Figures 5(a) and 5(b)), complying with the axon growth-inhibiting effects in sensory neurons (Figures 2(b) and 4(b)). These results show a strong correlation between the effect of epothilone B on axon growth and growth cone MTs.

### 3.4. Neurons of Different Type and Age Contain MTs That Differ in Stability

**Figure 7:**
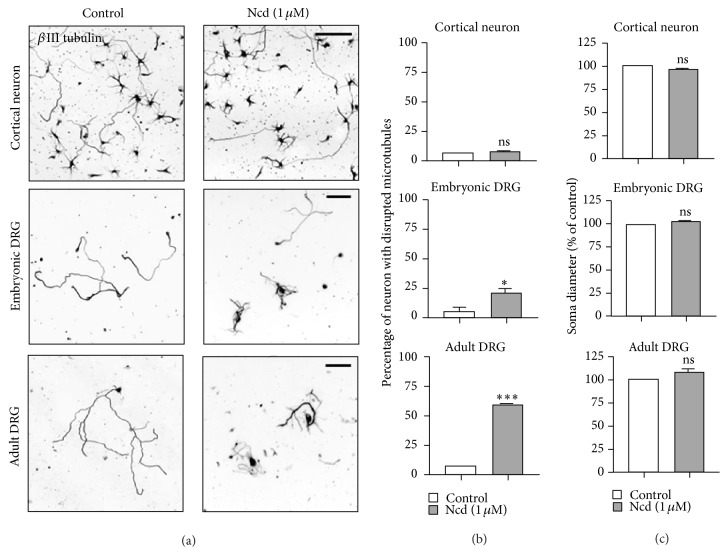
Nocodazole-induced degeneration in different types of neurons. (a–c) Embryonic (E15.5) cortical neurons, embryonic (E13.5) DRG neurons, or adult DRG neurons were cultured in the absence or presence of nocodazole (Ncd) (1 *μ*M, 30 min), as indicated. Neurons were immunostained with anti-Tuj1 antibodies. Representative images (a) and quantification of axon degeneration (b) and soma diameter (c) are shown. Scale bar, 50 *μ*m. ^*∗*^ *p* < 0.05 and ^*∗∗∗*^ *p* < 0.001; ns, statistically not significant.

We next wanted to understand why neurons of different type and age exhibit different susceptibility to epothilone. It is possible that different types of neurons contain MTs that differ in stability, which can affect responses to MSA. To compare MT stability among neurons, we treated the cells with the same concentration of nocodazole, a microtubule-depolymerizing drug, for the same duration, and then compared their ability to withstand. For quantitative assessment of changes in MT stability, we measured the mean fluorescence intensity of tubulin in the neurons treated with nocodazole or vehicle control. When neurons were exposed to 50 nM of nocodazole for 90 min, tubulin intensity was dramatically diminished in embryonic and adult DRG neurons, whereas persistent and strong signals were maintained in embryonic cortical neurons (Figures 6(a) and 6(b)). Consistent with the changes in fluorescence intensity, 50 nM of nocodazole inhibited axon growth in embryonic and adult DRG neurons but not in cortical neurons (Figure 6(c)). A closer examination of MTs revealed that MTs in sensory neurons (both adult and embryonic) became disrupted after nocodazole treatment, whereas axonal MTs remained intact in embryonic cortical neurons (Figures 6(d) and 6(e)). To induce more drastic depolymerization and axon degeneration, we treated neurons with a higher concentration of nocodazole (1 *μ*M) and compared their resistance. Axonal degeneration was evident only in embryonic and adult DRG neurons, with more dramatic degeneration observed in adult sensory neurons than embryonic sensory neurons (58.4 ± 1.9% versus 22.1 ± 2.4%). In striking contrast, cortical neurons were able to withstand nocodazole-induced degeneration and axons remained intact (Figures 7(a) and 7(b)). Of note, even in DRG neurons, degeneration occurred specifically in the axons and the diameter of cell bodies was unaffected (Figure 7(c)). Together, these results clearly show that sensory axons in the PNS contain less stable MTs that are prone to disruption and degeneration and suggest that neurons of distinct type and age contain MTs that differ in stability.

## 4. Discussion

MTs have been among the most successful targets for treating a number of malignancies. In addition, MSAs are emerging as promising therapeutic agents to treat neurodegenerative diseases and enhance axon regeneration after nerve injury. Systemic application of brain-penetrant MSAs in animal models of neurodegeneration and CNS injury prevented nerve degeneration [8, 9, 23, 24] and promoted axon regeneration [8], respectively. However, such treatment regimen of systemic application exposes MSA to the entire organism, and thus beneficial effects may come with tradeoffs. Considering the well-documented peripheral neuropathy and axon degeneration induced by MSAs that remain in the periphery due to poor BBB permeability [16], it is possible that the MSAs that cross the BBB cause CNS toxicity. Here we begin to tackle this issue by examining and comparing the possible cytotoxic effect of epothilone B, a brain-penetrant MSA, on CNS and PNS neurons.

The present study suggests that epothilone B can produce both beneficial and detrimental outcomes in culture, depending on not only the concentration of the drug but also the type of neurons being exposed, as seen in clinical settings. In all types of neurons tested, epothilone B exerted neurotoxicity at micromolar concentrations. Neurotoxicity was manifested as retardation of axon growth, and axon degeneration, as well as disorganization of MTs in the growth cone and the axonal shaft. However, at picomolar concentrations, epothilone promoted the growth of axons from young DRG and cortical neurons, while the same concentration drastically prevented axon growth from adult sensory neurons. Such dose-dependent studies clearly reveal that peripheral sensory neurons exhibit higher susceptibility as compared to cortical neurons and adult sensory neurons are more sensitive than their embryonic counterparts. It has been suggested that the selectivity of sensory axon degeneration in patients who have undergone chemotherapy might be due to the greater exposure of sensory fibers to MSAs, as many of such drugs do not cross the BBB and thus sparing CNS neurons from toxicity. According to this conjecture, fundamental degenerating aspects would commonly be induced in any of the types of neurons, if exposed, and thereby precludes the application of MSAs to treat injury and diseases in the CNS. However, here we show that sensory neurons are more susceptible to epothilone B even in culture, suggesting that the selective susceptibility of sensory neurons and chemotherapy-induced peripheral neurotoxicity reported in patients cannot be explained merely by more exposure, and other reasons might underlie the cell-type specific vulnerability. Results from our study suggest that sensitivity to MSA exposure reflects the stability of neuronal MTs, which is an intrinsic property of a neuron of particular type and age.

In adult DRG neurons, epothilone was inhibitory at all concentrations tested, in terms of both axon growth and viability (see Figure 4). Adult DRG culture has widely been exploited as an axon injury model, as long axons that already establish solid connections in an adult mouse should be severed at the first place prior to culture. Thus, the lack of axon growth-promoting effect in adult DRG neurons might be due to the preceding insult, that is, axonal injury, rather than the difference in MT stability. Indeed, axon injury is known to trigger a number of molecular changes, including the induction of regeneration-associated genes [25]. We, however, would like to place less emphasis on this because adult neurons were fixed at about 24 hr after plating, which is before regeneration-associated genes are fully induced. Moreover, we did not see any axon growth-promoting effect when various concentrations of epothilone were treated to adult DRG neurons isolated from conditioning lesioned mice at 7 days after sciatic nerve transection (data not shown). Therefore, we prefer the hypothesis that the hypersensitivity of adult DRG neurons to epothilone is attributed to the intrinsic vulnerability of MTs assembled in adult sensory neurons.

The present study suggests that the age of a neuron is a crucial factor that determines the susceptibility of a particular neuron to MSA exposure. To reach this conclusion, here we isolated neurons from mice of different ages and cultured them for the same duration. Alternatively, neurons obtained from mice of a certain age could be allowed to “age”* in vitro* by culturing them for different times. Culturing neurons for different times might recapitulate certain aspects of the ageing process that occurs* in vivo*. When embryonic or postnatal neurons are isolated, they first would undergo differentiation and then maturation, and the terminology of “ageing” can refer to one of the processes or both. At this point, it is unclear whether the stage of differentiation and the age of a neuron can be defined by a common set of parameters and whether the two processes are molecularly and functionally comparable. With respect to differentiation, some neurons are known to undergo stereotyped differentiation* in vitro*, enabling us to clearly mark the stage of differentiation morphologically and molecularly [5]. On the contrary, the process of ageing is not fully understood and there are no clear markers or milestones of neuronal ageing. Thus, it remains unknown the extent to which the ageing process that occurs* in vivo* is recapitulated when young neurons isolated from embryos or neonates are allowed to grow* in vitro*. Therefore, we think that the best way to compare the effects of epothilone on neurons of different ages is to actually isolate neurons from mice of different ages. To this end, we chose DRG neurons because these neurons could be prepared from embryos, neonates, and adult animals. When DRG neurons from embryonic, neonatal, and adult mice were cultured for the same duration and treated with epothilone following the same protocol, epothilone induced distinct effects (Figures 2 [Fig fig3]–4). These results provide strong support for our view that neuronal age is an important factor that determines the sensitivity of a neuron to epothilone B exposure.

In addition to neuronal type and age, differentiation stage might affect how neurons respond to MSA exposure. Investigating whether MT stabilization by MSA treatment would cause distinct effects on neurons in different differentiation stages would be a critical and interesting topic. This question might be more clearly addressed by applying MSAs to rat hippocampal neurons in a defined stage, as these neurons undergo well-documented, standardized stages of differentiation* in vitro* [26]. Further experiments are needed to interrogate the effects of differentiation and other factors on the consequences of MSA exposure.

Neuronal MTs have long been viewed as being very stable [13–15, 27], a property which is crucial to serve as solid tracks for intracellular transport and to maintain the complex morphological architecture of a neuron, which is, in most regions, irreplaceable in the existing circuitry of the adult mammalian CNS. However, the discovery and imaging of plus-end-tracking proteins (+TIPs) have revealed that a fraction of MTs is quite dynamic even in fully matured neurons [28, 29]. Neuronal MTs continuously undergo assembly and disassembly and are exquisitely sensitive to perturbations. Everexpanding body of evidence has suggested that a precise control of the assembly/disassembly of MTs is essential not only in the developing but also in the mature nervous system and that multiple layers of regulation exist to balance MT stability and dynamics. The exact mechanisms by which neurons fulfill the opposing needs are not completely understood, but substantial evidence supports that neuronal MTs are composed of stable and labile fractions that reside in distinct compartments in a neuron (e.g., axonal MTs are generally more stable than dendritic MTs) and even in each polymer (e.g., the stable and the dynamic regions are located toward the minus and the plus ends, resp.). By keeping distinct populations of neuronal MTs that differ in stability and dynamics, tasks can be specialized, for example, most stable MTs unique to neurons would play a role in maintaining the structural integrity, while dynamic MTs would provide the basis for neural plasticity and structural modifications in response to stimuli. It is possible that the proportion of stable (or dynamic) MTs is different depending on the type and age of a neuron. It is also possible that neurons of specific type and age express certain combinations of tubulin isoforms regulated in a distinct fashion, which is controlled by a myriad of microtubule-binding proteins, posttranslational modifications, and other cellular factors that affect tubulin assembly, modification, stability, and so forth. Thus, it is plausible that a neuron of a particular type and age harbors microtubules of unique biochemical and biophysical properties that control MT stability. Further experiments are required to understand why neurons of different type or age have different MT stability.

Our study suggests that when designing an MSA-based therapeutic regimen, it is of crucial importance to consider the type and age of a target neuron to be treated, as well as the concentration of the drug and exposure time. Studies aiming at testing the feasibility of applying BBB-penetrating MSAs for the treatment of nerve injury or neurodegenerative diseases should thoroughly examine whether such administration affects the intact nervous system, both the CNS and the PNS. In addition to neurons, glial cells have been implicated in the onset and progression of several neurodegenerative diseases [30] and in CNS injury [31, 32]. Although a large body of work has been devoted to promote neuroprotection and prevent nerve degeneration, much less has focused on glial biology and neuron-glia interactions. Before applying MSAs to treat diseases and injury in the CNS, future investigations should also thoroughly aim at examining the possible effects of MSAs on glia and neuron-glia interactions. To exploit MSAs to their full benefit and minimize unwanted effects in the PNS or elsewhere in the body, strategies should be devised to apply minimal effective concentration, limit the exposure time, and deliver MSAs directly to the site where needed. If peripheral neuropathy is indeed due to an intrinsic property of MTs in sensory neurons, systemic administration of epothilone B to treat CNS disorders should be avoided. Improved understanding of the mechanisms underlying MSA-induced peripheral neuropathy is essential not only for its prevention but also for the development of effective and safe agents to treat injury and diseases in the nervous system.

## Abbreviation

MSA: Microtubule-stabilizing agent
MT: Microtubule
CNS: Central nervous system
PNS: Peripheral nervous system
BBB: Blood-brain barrier
DRG: Dorsal root ganglion.
